# Improving Lung Cancer Education and Screening Navigation Skills Among HBCU Health Trainees: A Pilot Study of Program CONNECT

**DOI:** 10.1007/s13187-025-02704-0

**Published:** 2025-08-20

**Authors:** Shanada Monestime, Erika L. Thompson, Monica Wilson, Tamara McCants, Lovoria B. Williams

**Affiliations:** 1 GO2 for Lung Cancer, 2033 K Street NW, Suite 500, Washington, DC 20006 USA; 2 University of Texas Health Science Center at San Antonio, San Antonio, TX USA; 3 National University, San Diego, CA USA; 4 National Pharmaceutical Association, Phoenix, AZ USA; 5 College of Nursing, University of Kentucky, Lexington, KY USA

**Keywords:** Lung cancer disparities, Health professional trainees, HBCUs, Screening navigation, Health equity

## Abstract

Lung cancer is the leading cause of cancer death, disproportionately affecting Black communities due to delayed diagnoses and low screening rates. While these disparities persist, few educational interventions aim to equip health professional trainees—particularly those at Historically Black Colleges and Universities (HBCUs)—with the knowledge and confidence to address them. Empowering providers early in their training through culturally tailored education may help build a more prepared, equity-focused workforce. The purpose of this project was to evaluate a culturally tailored lung cancer education program, Program CONNECT, for health professional trainees at HBCUs. Program CONNECT was implemented in regions where lung cancer mortality among Black populations was ≥ 1.6 times higher than among White populations. From August to September 2024, healthcare trainees in nursing, medicine, pharmacy, and dentistry participated in a 90-min workshop covering lung cancer disparities, screening guidelines, and navigation strategies. A 21-item pre/post-test assessed changes in knowledge, beliefs, and self-efficacy. Participants (*N* = 100) were primarily nursing (63%), followed by pharmacy (29%), dentistry (5%), and medical (1%) students. While over half had healthcare experience, few had prior training in lung cancer navigation. Post-training, stigma related to personal responsibility for lung cancer significantly decreased (*p* = 0.005). Knowledge improved regarding risk factors, screening eligibility, disparities, and biomarker testing (*p* < 0.001). Confidence in identifying screening-eligible individuals, addressing barriers, and conducting difficult conversations improved significantly (*p* < 0.001). Recognition of ineligible screening scenarios increased, though shared decision-making knowledge remained unchanged. Interest in pursuing a cancer-related specialty rose from 66 to 78% (*p* < 0.001). Program CONNECT significantly improved knowledge and navigation self-efficacy among HBCU health trainees. Expanding this training across HBCUs may strengthen oncology workforce diversity and promote more equitable lung cancer outcomes in high-disparity communities.

## Introduction

Lung cancer (LC) stands as a devastating public health crisis, claiming more American lives annually than breast, pancreatic, and prostate cancers combined, with an estimated 124,730 fatalities each year [1]. While national incidence and mortality rates have declined in recent years, these improvements have not been equitably experienced. Black communities continue to face disproportionately high LC mortality, shaped by lower lung cancer screening (LCS) uptake, delayed diagnoses, and persistent systemic barriers [2–4]. These disparities are especially pronounced in places like Washington, D.C. and East Baton Rouge Parish, Louisiana. In D.C., Black residents experience LC incidence and mortality rates that are approximately 2 to 2.9 times higher than those of Whites [5–7]. In East Baton Rouge, disparities are most severe among Black males, who face incidence and mortality rates about 1.5 to 1.7 times higher than their White counterparts [6]. The journey to diagnosis is often fraught with delays for Black Americans, who are 15% less likely to receive an early-stage diagnosis compared to White patients, frequently presenting with significantly more advanced disease [3, 4]. Delayed diagnosis severely limits treatment options and significantly reduces survivorship, especially given that nearly half of all LC’s are diagnosed at an advanced stage, when survival rates are lowest [2].

Compounding these clinical disparities is a striking lack of racial diversity among the oncology workforce. Black oncologists comprise a mere 2–3% compared to 13% of the U.S. population—a gap that further perpetuates these inequities by limiting culturally competent care and hindering the crucial development of patient-provider concordance [8, 9]. Despite the urgency to dismantle these disparities, a critical void persists in the landscape of healthcare education: the conspicuous absence of culturally tailored interventions designed to equip future healthcare providers with the specialized knowledge and skills required to effectively navigate and mitigate LC inequities. While a growing body of research spotlights patient-focused interventions aimed at bolstering health literacy and engagement, surprisingly few studies have rigorously evaluated education programs for healthcare providers or trainees, particularly within institutions that predominantly serve Black communities or Historically Black Colleges and Universities (HBCUs) [10, 11]. This oversight is particularly concerning given that comprehensive LC education is frequently either entirely missing or woefully insufficient within the core health curricula across diverse disciplines such as nursing, pharmacy, and medicine [12]. This educational lacuna leaves a generation of emerging health professionals inadequately prepared to confront the intricate challenges of LC care and the deeply embedded disparities that plague its delivery.

This project confronts these critical deficiencies by rigorously implementing and evaluating a targeted, culturally relevant educational intervention within the unique and vital ecosystem of HBCUs. By strategically focusing on these institutions, this initiative harnesses the inherent strength of HBCUs in fostering diverse talent and aims to develop a generation of healthcare professionals attuned to health equity in LC. To achieve this, we developed Program CONNECT (**C**oordinate, **O**rganize, and **N**avigate **N**ecessary **E**quitable **C**are for **T**hose at Risk for or Diagnosed with Lung Cancer), a 90-min educational intervention. The primary objective was to evaluate the intervention’s impact on participants’ knowledge, beliefs, and self-efficacy related to lung cancer care—including screening and navigation—and to assess their interest in cancer-focused careers among a predominantly Black trainee population.

## Methods

### Study Setting and Design

This prospective one-group pretest–posttest quasi-experimental study was conducted in Washington, D.C., and Baton Rouge, Louisiana. Educational sessions took place at Howard University and Southern University A&M College—both HBCUs with health professional training programs in either pharmacy, nursing, medicine, and dentistry. The study evaluated the impact of a 90-min educational intervention on participants’ LC knowledge, beliefs, and navigation-related self-efficacy using pre- and post-intervention surveys. The WCG Institutional Review Board approved the study. All participants provided electronic informed consent prior to participation.

### Participants and Recruitment

The target sample size was 100 participants across both sites. Inclusion criteria were as follows: (1) students affiliated with one of the two partnering institutions, (2) enrolled in medicine, pharmacy, nursing, or dentistry, (3) in training throughout the intervention period, and (4) completed both the pre- and post-intervention surveys. Recruitment was conducted via class announcements, faculty referrals, and printed flyers, and campus-wide emails. One of the participating sites was a nursing-only institution, while the second site offered multiple health professional programs (e.g., medicine, pharmacy, dentistry, and nursing). Although outreach efforts were inclusive of all disciplines at the multi-program site, nursing students were more likely to participate due to a course credit incentive offered by their faculty. No such credit was extended to other departments, which may have impacted cross-disciplinary participation.

### Intervention and Data Collection Procedures

The educational intervention was adapted from a previous study by reformatting the four-session intervention to a scripted 90-min PowerPoint [13, 14]. The session began with a brief overview of learning objectives, followed by the administration of a pre-evaluation survey. The workshop consisted of five sequential instructional modules covering LC disparities, treatment options, risk factors, screening guidelines, and screening navigation with an emphasis on motivational interviewing. Details on each module’s content and learning objectives are presented in Table 1.

**Table 1:** Workshop modules and learning objectives

Module	Title	Learning objectives
Module 1	Understanding Lung Cancer Disparities	- Define cancer and lung cancer incidence
		- Describe racial and geographic disparities
		- Explain stage at diagnosis and associated survival rates
		- Recognize systemic contributors to disparities (e.g., social determinants, marginalization)
Module 2	Lung Cancer Treatment Options	- Summarize standard treatments (surgery, chemo, radiation, immunotherapy, etc.)
		- Introduce precision medicine and biomarker testing
		- Discuss the importance of timely and tailored treatment
Module 3	Lung Cancer Risk Factors	- Identify major risk factors (e.g., smoking, radon, pollution)
		- Understand how smoking causes lung cancer
		- Discuss prevalence and behavioral contributors to risk
Module 4	Lung Cancer Screening	- Explain low-dose CT and its importance
		- Identify eligibility criteria and pack-year calculation
		- Review barriers to screening (knowledge, stigma, access)
		- Understand screening frequency and safety
Module 5	Lung Cancer Screening Navigation	- Practice motivational interviewing techniques to engage patients
		- Guide patients through lung cancer screening eligibility checks and navigation steps
		- Use empathy and active listening to address resistance and stigma
		- Support informed, patient-centered decision-making

Instruction was delivered in person by three content experts: one specializing in lung cancer, one in tobacco-related risk factors, and one in motivational interviewing. Sessions included large-group presentations followed by peer discussions and case-based simulations. Participants were paired during the interactive components to encourage engagement and application of knowledge. Two case scenarios were used to assess knowledge of eligibility for screening. A facilitator guide was used to ensure consistency across both locations. A facilitator guide was used to ensure fidelity across locations. Surveys were administered immediately before and after the intervention using Qualtrics or paper forms. The pre-test included demographics: age, gender, race, ethnicity, discipline, year of training, years of healthcare experience, prior LC training, and location. Pre-post surveys aligned with the learning objectives for each module. An existing validated instrument did not exist that would measure all relevant key knowledge, attitudes, and beliefs; thus, we developed an instrument by aligning questions with training content, adapting some items from existing knowledge scales [15], and receiving input from the GO2 for Lung Cancer Community Engaged Research Advisory Board. Self-efficacy items were adapted from Ahmed et al. and measured confidence in identifying patients eligible for LCS, helping clients overcome barriers to LCS, LCS navigation skills, and having difficult conversations about LCS. Participants also indicated interest in lung cancer advocacy and cancer-related careers.

### Data Analysis

Descriptive statistics (means, standard deviations, frequencies) were used to summarize participant demographics. Knowledge items were scored as correct or incorrect; self-efficacy and attitude items were measured using Likert-type scales. Due to distribution, 5-point Likert responses for self-efficacy variables were collapsed into a binary variable (not/slightly confident vs. somewhat/very confident). Paired *t*-tests were used to assess pre/post changes in continuous variables. To account for the paired data, McNemar’s test was used to evaluate changes in binary variables, and McNemar-Bowker test was used to evaluate changes in categorical variables. All analyses were conducted using SPSS.

## Results

### Participant Characteristics

A total of 100 participants were included in the analysis. Table 2 provides the sample details; briefly, the mean age was 24.6 years (SD = 3.3); the majority (97%) were Black/African American and female (85%). Approximately 25% reported having a friend or family member with LC. Most were from nursing (63%) and were in their last year of training. Over half (52%) reported some form of prior healthcare experience, but only four participants had received previous training related to LC navigation.

**Table 2:** Lung cancer workshop participant demographics (*n*** = **100)

Characteristic	Mean
Age *	24.6 years mean (SD=3.3)
	*N*
*Gender*	
Female	85
Male	15
*Race*	
Black/African American	97
Other	1
White	2
Hispanic	4
*Friend/family with lung cancer*	
No	65
Not sure	11
Yes	24
*Discipline*	
Medicine	1
Nursing	63
Pharmacy	29
Dentistry	5
Other	2
*Year*	
1^st^	6
2^nd^	16
3^rd^	19
4th	48
Other	11
*Years Healthcare Experience*	
0	42
1-2	30
More than 3	22
Missing	6
*Prior Lung Cancer Training*	
Yes	4
Not sure	4
Missing	1
No	91
*Location*	
Baton Rouge	65
Washington, DC	35

*Missing 1

### Beliefs

The results of the pre-and post-survey are included in Table 3. Of the 100 participants, at baseline, 94% responded that individuals diagnosed with LC were responsible for their condition to some degree due to lifestyle choices. Post-intervention, the proportion attributing any level of responsibility decreased to 75%, and those reporting no responsibility increased to 25%. This change in belief distribution was statistically significant (*p* = 0.005).

**Table 3:** Pre-post comparison among participants in a lung cancer workshop (*n*** = **100)

Characteristic	Pre, %	Post,%	*P*-value
*Beliefs*			
To what extent do you believe that individuals diagnosed with lung cancer are responsible for their condition due to lifestyle choices?			0.005
Completely responsible	2	4	
Mostly responsible	29	16	
Slightly responsible	63	55	
Not responsible at all	6	25	
*Knowledge*			
Most new lung cancer diagnoses are_______.			<0.001
I don’t know	29	0	
Stage 1	9	4	
Stage 2	23	0	
Stage 3	33	8	
Stage 4^	6	88	
Which cancer is the leading cause of cancer death for men and women in the United States?			<0.001
Breast	12	1	
Colon	12	5	
I don’t know	2	0	
Lung^	71	94	
Prostate	3	0	
What is meant by ’cancer disparities’ in the context of lung cancer screening and outcomes?			0.003
Differences in how lung cancer is perceived and addressed in public health campaigns.	6	2	
Factors that influence the variety of lung cancer symptoms experienced by patients.	8	4	
The variation in types of lung cancer detected through screening across different demographic groups.	10	4	
Unequal access to lung cancer screening, treatment, and outcomes, due to socioeconomic, racial, or geographic factors.^	76	90	
Nicotine causes lung cancer.			<0.001
False^	9	78	
True	84	21	
Unsure	7	1	
Only smokers can get lung cancer.			n/a
False^	100	99	
What is the second leading cause of lung cancer?			<0.001
Air pollution	21	4	
Family history	19	1	
I don’t know	8	0	
Radon^	1	92	
Secondhand smoke	51	3	
Eligibility age 50-80 years old			<0.001
No	10	3	
Unsure	32	0	
Yes^	58	97	
Eligibility smoke at least 1 pack per day for 20 years			0.003
No	10	3	
Unsure	9	1	
Yes^	81	96	
Eligibility current smoker*			0.648
No	8	10	
Unsure	8	2	
Yes^	84	87	
Eligibility past smoker within last 15 years*			0.243
No	16	16	
Unsure	10	1	
Yes^	74	82	
Eligibility has private insurance*			0.296
No^	40	47	
Unsure	25	18	
Yes	35	34	
Eligibility never smoked*			<0.001
No^	41	96	
Unsure	28	0	
Yes	31	3	
What are the recommended screening methods for lung cancer?			<0.001
Missing	2	0	
Chest X-Ray	24	3	
Low-Dose Computed Tomography (LDCT)^	55	95	
MRI	11	1	
PET scan	8	1	
How often should a person get screened for lung cancer?			<0.001
Every 2 years	25	3	
Every 5 years	31	2	
Every year^	24	93	
I don’t know	17	0	
Once	3	2	
When should someone stop being screened for lung cancer?			0.332
I don’t know or incorrect	98	85	
They quit smoking more than 15 years ago; They have other health problems that may shorten their life; They are not able or willing to be treated for lung cancer^	2	15	
Getting a biomarker test can help identify clinical trials and new treatments that are not yet available on the market.*			<0.001
False	4	3	
True^	70	90	
Unsure	26	6	
*Knowledge Application*			
Leonna is 47 years old and has smoked half a pack a day for 20 years. Should she get screened for lung cancer?			<0.001
I don’t know	1	1	
No^	2	79	
Yes	97	20	
Raymond is 75 years old and quit smoking 5 years ago. When he did smoke, he smoked two packs a day for 20 years. Should he get screened for lung cancer?			0.687
I don’t know	1	1	
No	1	3	
Yes^	98	96	
Part of shared decision-making is assessing the person’s values and preferences. In the case of lung cancer screening, what is a way to start this part of the conversation?*			0.860
Are you scared about finding cancer?	4	4	
As you think about lung cancer screening, is early detection important to you?	42	50	
As you think about lung cancer screening, what’s important to you?^	40	42	
Do you want to know if you have lung cancer?	3	2	
I don’t know	11	1	
*Self-Efficacy*			
How confident are you in identifying individuals who should be screened for lung cancer?**			<0.001
Not at all to slightly confident	61	5	
Somewhat to very confident	38	94	
How confident do you feel about your ability to help individuals overcome barriers to lung cancer screening?*			<0.001
Not at all to slightly confident	45	8	
Somewhat to very confident	55	91	
How confident do you feel about your ability to help people navigate individuals to get screened for lung cancer?*			<0.001
Not at all to slightly confident	41	4	
Somewhat to very confident	59	95	
How confident do you feel about your ability to have difficult conversations with people resistant to lung cancer screening?*			<0.001
Not at all to slightly confident	46	12	
Somewhat to very confident	54	87	
*Interest*			
How interested are you in advocating for better support and resources for lung cancer patients?*			0.377
Not interested at all	2	2	
Slightly interested	8	3	
Somewhat interested	27	24	
Very interested	63	70	
How interested are you in pursuing a specialty focused on cancer?*			<0.001
Not interested at all	16	8	
Slightly interested	28	13	
Somewhat interested	41	40	
Very interested	15	38	

^Correct Response and items with a correct response compared correct vs. incorrect for statistical testing; *Missing 1 on post-test; **Missing 1 on pre- and post-test

### Knowledge

Post-intervention results showed statistically significant improvements in lung cancer knowledge across multiple domains (all *p* < 0.001 unless noted). The most notable gains included correct identification of stage at diagnosis (6% pre vs. 88% post), radon as the second leading cause of lung cancer (1% vs. 92%), and clarification that nicotine is not a direct cause (9% vs. 78%). Understanding of screening criteria improved substantially—for example, correct identification of the screening method (LDCT) rose from 55 to 95%, and knowledge of screening frequency (annually) increased from 24 to 93%. Awareness of the purpose of biomarker testing also improved (70% to 90%). No meaningful changes were observed for shared decision-making prompts (40% vs. 42%, *p* = 0.860) or for questions where baseline knowledge was already high (e.g., 99% correctly rejected the myth that only smokers get lung cancer).

### Self-Efficacy

Participants reported statistically significant improvements in self-efficacy related to LC screening navigation following the intervention. The percentage of participants who felt somewhat to very confident in identifying individuals eligible for screening increased from 38 to 64% (*p* < 0.001). Confidence in helping individuals overcome barriers to screening increased from 55 to 91% (*p* < 0.001), and confidence in navigating individuals to screening rose from 59 to 95% (*p* < 0.001). Confidence in having difficult conversations with individuals resistant to screening also improved, increasing from 54 to 87% (*p* < 0.001).

### Interest

Prior to the training, 90% of participants expressed being somewhat or very interested in advocating for increased support and resources for lung cancer patients. This increased slightly to 94% following the intervention, though the change was not statistically significant (*p* = 0.377). Interest in pursuing a specialty related to cancer care increased significantly, with the proportion of participants reporting they were very interested rising from 15 to 38%, and overall interest (somewhat or very) increasing from 66 to 78% (*p* < 0.001).

## Discussion

This study evaluated the impact of Program CONNECT—a culturally tailored, 90-min educational intervention—on lung cancer knowledge, beliefs, self-efficacy, and career interest among health professional trainees at two HBCUs. Results showed significant and meaningful improvements across all domains, including increased knowledge of screening guidelines, greater confidence in navigating patients through the screening process, and reduced stigmatizing beliefs. Participants also reported greater confidence in guiding patients through the screening process and communicating effectively, with many expressing heightened interest in cancer-related careers. These findings are particularly noteworthy given the early stage of training among participants and the unique setting of HBCUs, which serve as important access points for reaching historically underrepresented populations in medicine.

Several educational interventions at HBCUs have focused on cancer-related topics, particularly HPV prevention, cervical cancer screening [16–19], tobacco-related behaviors [20], and cancer genetics [21]. These programs, like ours, leveraged culturally informed content to improve knowledge and shift attitudes among Black college students—affirming the value of trusted institutions in advancing health equity. However, most prior efforts emphasized general cancer awareness or behavior modification, rather than preparing trainees with practical knowledge and applied skills to address lung cancer specifically. In contrast, Program CONNECT focused on a leading yet often overlooked cause of cancer death—lung cancer—and uniquely targeted students earlier in their clinical trajectory. This distinction is critical, as our intervention not only enhanced awareness but also aimed to build confidence in screening navigation and patient engagement.

Moreover, while prior initiatives like SPARCC [22] and the CaRE(2) Health Equity Center [23] aimed to increase workforce diversity through intentional recruitment, mentoring, and research opportunities, Program CONNECT was not explicitly designed to promote oncology career interest. However, several students reported a greater interest in working with patients at risk for lung cancer or pursuing careers tied to cancer prevention and education. Culturally relevant exposure during training may help generate early interest in career-related fields, even in the absence of structured mentorship. While these findings are encouraging, additional research is needed to assess whether such interest leads to long-term career engagement in oncology. Embedding cancer education within trusted institutions like HBCUs may be a promising step toward addressing persistent underrepresentation of Black providers in oncology and cancer research [22, 24].

In addition to shifts in career interest, quantitative findings suggested that Program CONNECT prompted critical reflection on lung cancer stigma and improved understanding of nuanced topics. For example, fewer participants attributed blame to individuals diagnosed with lung cancer post-intervention, indicating that intentional, culturally grounded education may help reduce bias and foster empathy. Knowledge gains also extended to underemphasized areas such as radon exposure, screening eligibility, and biomarker testing—topics aligned with national guidelines and essential to ensuring timely, evidence-based care.

This study has several strengths. It was implemented across two geographically distinct HBCUs, ensuring contextual and regional relevance. The use of a matched pre/post-test design enabled a rigorous evaluation of individual change. It also engaged a historically underrepresented population in lung cancer research and education—an area that remains chronically understudied. However, limitations must be acknowledged. The absence of long-term follow-up prevents us from assessing whether improvements were sustained over time or translated into clinical behaviors. Outcomes were self-reported, which may introduce bias. Generalizability may be limited to similar institutions, and a small subset of participants had prior exposure to lung cancer content, though this likely attenuated, rather than exaggerated, the intervention’s observed effects. Our sample was predominantly female, which likely reflects the gender makeup of the two largest participant groups—nursing and pharmacy students—who are nationally 88% and 60–62% female, respectively [25, 26]. This demographic imbalance may limit applicability to more gender-diverse health professional training setting.

Future directions for Program CONNECT include both expanded implementation and continued research. Scaling will involve increasing and establishing institutional partnerships in regions with HBCUs that experience disparities in lung cancer incidence and/or mortality in order to deliver this intervention. For institutions that do not meet this criterion, we will prioritize developing a faculty toolkit to support independent implementation. The next phase of the program will involve hiring and training students who completed the intervention to serve as lung cancer screening navigators within their communities. These student navigators will support outreach, education, and linkage to screening—applying the knowledge and skills gained through Program CONNECT to address real-world disparities. Additional evaluation is needed to explore how this intervention can be integrated into health education curricula and to examine its long-term influence on participants’ professional trajectories, including oncology-related rotations, research involvement, and post-graduate engagement in cancer care and advocacy.

## Conclusion

Program CONNECT demonstrated that a brief, culturally tailored educational intervention can significantly improve lung cancer knowledge, reduce stigma, and increase self-efficacy among health professional trainees at HBCUs. Notably, the intervention also sparked increased interest in cancer-related careers, even though this was not an explicit aim. These outcomes highlight the potential of targeted, culturally responsive education to prepare future providers to address persistent disparities in LC screening and care. Integrating similar programs into professional training may be a critical step toward building a more informed, diverse, and equity-driven healthcare workforce. Continued investment in early, relevant exposure is essential to ensure that those most impacted by lung cancer receive timely, compassionate, and effective support.

## Data Availability

The aggregated, de-identified data supporting the findings of this study are available from the corresponding author upon reasonable request.
